# Lumbar Roll Usage While Sitting Reduces the Forward Head Posture in Healthy Individuals: A Systematic Review with Meta-Analysis

**DOI:** 10.3390/ijerph18105171

**Published:** 2021-05-13

**Authors:** Yusuke Handa, Kenya Okada, Hiroshi Takasaki

**Affiliations:** 1 Graduate School of Rehabilitation Science, Saitama Prefectural University, Koshigaya 343-8540, Japan; 2381305q@spu.ac.jp; 2 Department of Rehabilitation, Kansai Rehabilitation Hospital, Toyonaka 560-0054, Japan; kenya220@icloud.com; 3 Department of Physical Therapy, Saitama Prefectural University, Koshigaya 343-8540, Japan

**Keywords:** alignment, lumbar roll, posture, sitting

## Abstract

This systematic review and meta-analysis investigated whether the use of a lumbar roll reduced forward head posture (FHP) while sitting among individuals with or without musculoskeletal disorders. EMBASE, MEDLINE, and the Cochrane Library were systematically searched from their inception to August 2020. The quality of evidence for variables used in the meta-analysis was determined using the GRADE system. Five studies satisfied the criteria for data analysis. All studies included individuals without any spinal symptoms. Data from five studies on neck angle showed a statistically significant (*p* = 0.02) overall effect (standardized mean difference (SMD) = 0.77), indicating a lesser neck flexion angle while sitting with a lumbar roll than without it. Data from two studies on head angle showed a statistically significant (*p* = 0.04) overall effect (SMD = 0.47), indicating a lesser head extension angle while sitting with a lumbar roll than without it. In each meta-analysis, the quality of evidence was very low in the GRADE system. The use of a lumbar roll while sitting reduced FHP among individuals without spinal symptoms.

## 1. Introduction

Optimal cervical lordosis contributes to the efficient distribution of the heaviest head load in the body to the anterior and posterior elements of the cervical spine. Flattening of the cervical spine, which results from a forward head posture (FHP) [1], increases the compressive force on the anterior vertebral element and increases the tensile force on the posterior vertebral element. Prolonged FHP is a contributing factor for the development of neck and shoulder problems [2,3]. Tension-type and posture-related headaches have been correlated with the magnitude of FHP [4,5,6]. Therefore, prolonged FHP should be avoided to prevent headaches, as well as neck and shoulder problems.

Studies have shown that sitting promotes greater FHP compared to standing [7] and postural correction while sitting to achieve lumbar lordosis reduces FHP [8]. However, ergonomically designed chairs that maintain lumbar lordosis are not always used. As such, a portable lumbar roll can be a convenient tool that helps achieve and maintain lumbar lordosis and reduce FHP during prolonged sitting given its accessibility.

Some studies investigating the effects of lumbar roll use on neck and/or head posture have reported that the use of a lumbar roll significantly influenced neck and/or head angles [9,10], whereas others have not found such findings [11,12]. Therefore, a systematic review and meta-analysis, which has been unavailable to date in the EMBASE, MEDLINE, and the Cochrane Library, is necessary to investigate whether the use of a lumbar roll reduces FHP while sitting. Including not only randomized controlled trials (RCTs) but also cross-sectional studies was considered prudent given that the availability of a sufficient number of RCTs for meta-analysis remains unknown.

The current study aimed to investigate whether the use of the lumbar roll reduced the FHP while sitting in individuals with or without musculoskeletal disorders through a systematic review with meta-analysis of RCTs and cross-sectional studies.

## 2. Materials and Methods

### 2.1. Identification and Selection of Studies

The current review was registered a priori with the PROSPERO (CRD42019127104). The first author (H.T.) performed a systematic search of the following databases, all of which have been recommended in a guideline for the database in systematic reviews of musculoskeletal disorders [13] from their inception to 31 August 2020: EMBASE, MEDLINE, and the Cochrane Library, using the search terms outlined in Supplementary Table S1. We undertook cross-referencing through discussions with a panel of six experts, and manually searched for relevant literature cited in the studies included herein.

Our study’s inclusion criteria were based on the PICOS framework and included the following: healthy individuals or individuals with musculoskeletal disorders, use of a lumbar roll, comparison between the absence and presence of a lumbar roll, neck or head angle while sitting or horizontal displacement of the head while sitting, and published journal articles or thesis and experimental study design. Exclusion criteria were as follows: use of interventions other than a lumbar roll, comparison between different chairs, and insufficient data for meta-analysis (i.e., missing data for the number of participants and/or means and standard deviations). When insufficient data were available for calculating the standardized mean difference (SMD), attempts were made to contact the corresponding author in order to obtain the data. Contact was attempted twice, with the second contact being a single reminder sent one week after the first contact. No language restriction was established.

Two authors (Y.H. and K.O.) independently screened the literature by reviewing the titles and abstracts without blinding of author names. To determine which studies to include in the analyses, both authors independently performed a full-text inspection of studies that either author had remained after their screening. Discrepancies in the full-text inspection were settled by another author (H.T.).

### 2.2. Assessment of the Studies’ Characteristics

Two authors (Y.H. and K.O.) independently examined the methodological quality of the included studies, with disagreements between them being resolved by another author (H.T.). Agreement between the two authors (Y.H. and K.O.) on methodological quality was examined using Cohen’s kappa and % agreement with the following kappa values: <0.4 = poor agreement, 0.41–0.60 = moderate agreement, 0.61–0.80 = good agreement, and 0.81–1.0 = very good agreement [14].

The studies’ methodological quality was assessed using the modified McMaster Critical Review Form for Quantitative Studies [15], where a total score ranges from 0 to 16. This critical appraisal tool was selected given that: (1) it has acceptable inter-examiner reliability as reported in several studies [15,16,17], (2) most of the 16 points are covered in the CONSORT statement and were considered comprehensive, (3) it can be used for not only RCTs but also cross-sectional studies, and (4) a threshold of poor quality has been used in previous studies [15,16,17]. The present study followed the modified guidelines for the critical appraisal tool established in a previous study to enhance inter-examiner agreement [15]. However, ambiguities existed in the interpretation of the two criteria for validity and reliability of outcomes, for which we have added additional criteria to enhance inter-examiner agreement: published evidence is not required (validity of outcomes); when the evidence for reliability is explained with references, the criterion is satisfied when evidence for reliability is clearly shown in similar participants (reliability of outcomes). Studies with a score of ≤8 in the modified McMaster Critical Review Form for Quantitative Studies (poor quality) were excluded from data synthesis. A threshold of 8 was selected based on previous studies [15,16,17,18] and was considered reasonable given that it is the middle point of the whole scale.

The quality of evidence for variables used in the meta-analysis was determined using the Grading of Recommendations Assessment, Development, and Evaluation (GRADE) system [19], which has five criteria: risk of bias, imprecision, inconsistency, indirectness, and publication bias. This system determines quality as high, moderate, low, or very low [20]. Given that the present study focused on cross-sectional comparisons of a lumbar roll usage while sitting, the quality of evidence was downgraded from low-quality evidence. No upgrading was present when downgrading was performed in any of the five criteria [21]. The following criteria utilized in previous studies [15,16,17] were used for downgrading: (1) imprecision where meta-analysis included <200 participants in each arm (one level) or <100 participants in each arm (two levels) [22], (2) inconsistency where the I^2^ value for heterogeneity was more than 75% [22], and (3) indirectness where clinically different populations or outcome measures or indirect comparisons were included in the meta-analysis. Downgrading by two levels was used for conservative consideration when any of the three criteria were satisfied [23]. Publication bias was identified when the results of the meta-analysis came from several small studies or when the meta-analysis included studies sponsored by the industry [24]. Finally, the lowest quality of evidence among the five criteria was used as the quality of evidence for each variable included in the meta-analysis. Two authors (Y.H. and K.O.) independently provided GRADE ratings, with disagreement between them being settled by another author (H.T.).

### 2.3. Data Analysis

Two authors (Y.H. and K.O.) independently extracted quantitative data of the neck or head angle or horizontal displacement of the head and qualitative data based on the PICOS framework. The extracted quantitative data included sample size, important eligibility criteria, age, and gender, lumbar roll information and backrest angle, measurement time points, measures, measurement tools, and other outcome measures not included in the current review, and study design, as well as the source of funding. Any disagreement between the two authors regarding the extracted data was settled by another author (H.T.).

When similar outcomes for neck or head angle or horizontal head displacement were obtained, a meta-analysis using Review Manager 5 (The Nordic Cochrane Centre, København Ø, Denmark) was performed. Using a random-effects model, we calculated the SMD and its 95% confidence intervals (CIs). The I^2^ index was assessed to determine the magnitude of between-study heterogeneity in the meta-analysis, with I^2^ values of 25%, 50%, and 75% indicating low heterogeneity, moderate heterogeneity, and high heterogeneity, respectively [25]. Post-hoc subgroup analyses were attempted when high heterogeneity or multiple RCTs were present.

When data across multiple conditions were available for the meta-analysis, we used: (1) those that were closest to the backrest angle of 110°, given that this is the most effective angle to change neck and head posture using a lumbar roll [10], and (2) those immediately after the use of a lumbar roll.

## 3. Results

### 3.1. Flow of Study Selection

**Figure 1:**
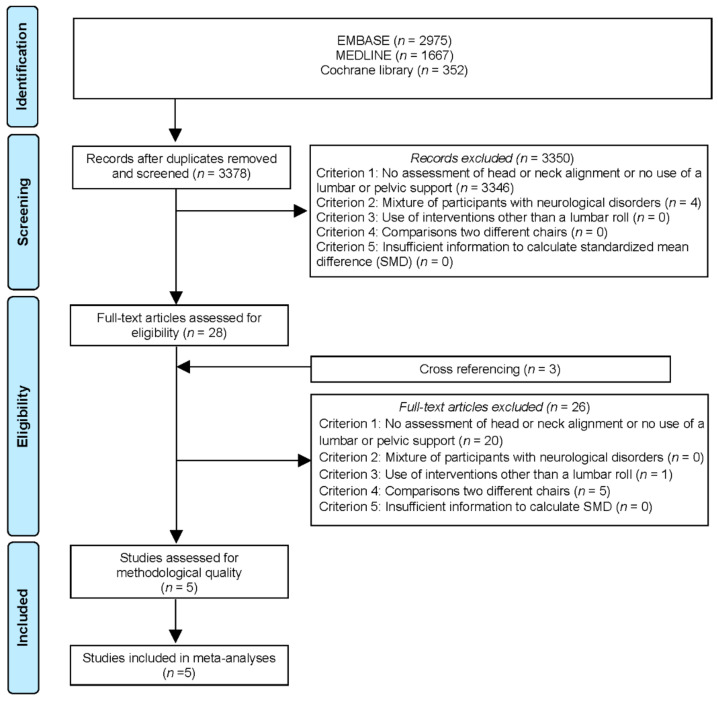
Flow of study selection.

The flow of the study selection is shown in Figure 1, which followed the PRISMA guidelines. The two authors disagreed on the exclusion of seven studies (agreement of exclusion = 99.8%) during the screening process. No disagreement of exclusion occurred between the two authors during the full-text review. Five studies [9,10,11,12,26] were examined for methodological quality, all of which were included in the analysis. Supplementary Table S2 presents a list of the 31 included studies in the full-text review.

### 3.2. Characteristics of Studies

Table 1 presents a summary of the five studies [9,10,11,12,26]. No disagreement occurred during data extraction. All participants included in the studies had no spinal symptoms. Table 2 presents the scores for the modified McMaster Critical Review Form for Quantitative Studies. One study [9] was written in Korean. There was moderate agreement on methodological quality between the two authors: kappa (*p*-value, 95% CIs) = 0.59 (*p* < 0.001, 0.36–0.82), and % agreement = 75%. The methodological faults observed in at least 80% of the studies concerned study design (Criterion 3), blinding (Criterion 4), and sample size (Criterion 6).

#### Effect of Intervention

**Figure 2:**
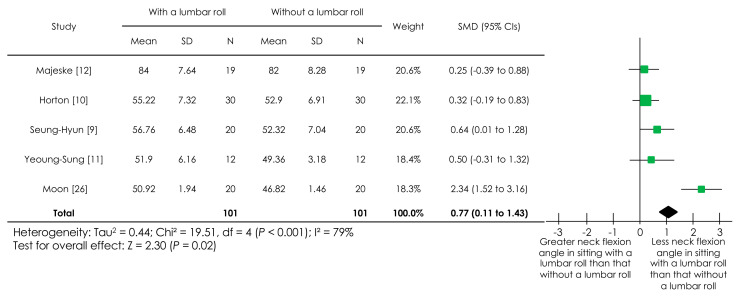
Forest plot of the effect of a lumbar roll in sitting on a neck angle. Abbreviations: SMD, standardized mean difference; CIs, confidence intervals.

With regard to neck angle, four studies [9,10,11,12,26] reported on the cranio-vertebral angle, that is, the angle between the horizontal line through the spinous process of C7 and the line from the spinous process of C7 through the tragus of the ear. Another study [12] reported on the angle between the horizontal line through the acromion and the line from the acromion through the tragus of the ear. Given the similarity between these two neck angles and the use of the SMD, a meta-analysis of the five studies [9,10,11,12,26] was performed, a forest plot of which is presented in Figure 2. There was a statistically significant overall effect (*p* = 0.02), indicating that sitting with a lumbar roll promoted a lesser neck flexion angle than sitting without it. The I^2^ value was 79%, indicating high heterogeneity.

**Figure 3:**
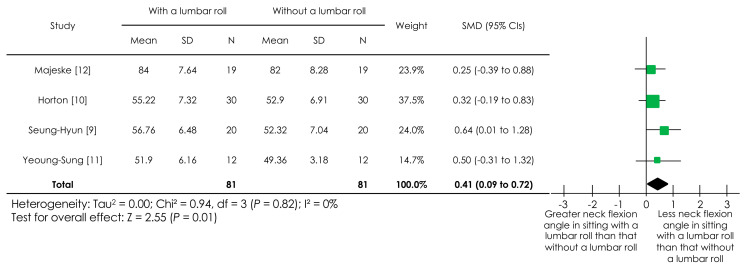
Post-hoc forest plot of the effect of a lumbar roll in sitting on a neck angle, excluding individuals with a cranio-vertebral angle < 51°. Abbreviations: SMD, standardized mean difference; CIs, confidence intervals.

A post-hoc meta-analysis was conducted after excluding one RCT [26] (Figure 3) given that the RCT included individuals with a cranio-vertebral angle < 51°, whose SMD value seemed far larger than the SMD values in the other four studies [9,10,11,12]. The cumulated sample size was 81 participants in each group, with a statistically significant overall effect having been observed (*p* = 0.01, SMD (95% CIs) = 0.41 (0.09–0.72)), indicating that sitting with a lumbar roll promoted a lesser neck flexion angle than sitting without it. The I^2^ value was 0%, indicating low heterogeneity.

**Figure 4:**
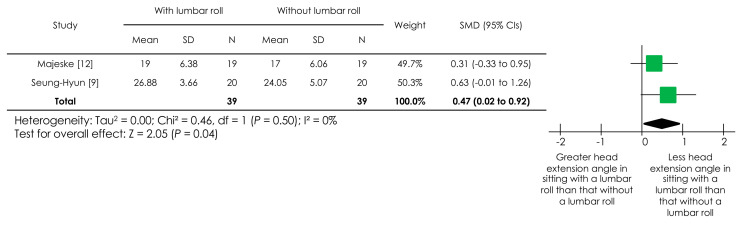
Forest plot of the effect of a lumbar roll in sitting on a head angle. Abbreviations: SMD, standardized mean difference; CIs, confidence intervals.

With regard to head angle, two studies [9,12] reported the angle between the horizontal line through the tragus of the ear and the line from the tragus of the ear through the eye. Another study [11] reported on the angle between the line from the spinous process of C7 through the tragus of the ear and the line from the tragus of the ear through the eye. The corresponding author of the study provided no additional data on the angle between the horizontal line through the tragus of the ear and the line from the tragus of the ear through the eye [11]. Thus, a meta-analysis was conducted using only two studies [9,12], a forest plot of which is presented in Figure 4. There was a statistically significant overall effect (*p* = 0.04), indicating that sitting with a lumbar roll promoted a lesser head extension angle than sitting without it. The I^2^ value was 0%, indicating low heterogeneity.

Table 3 demonstrates the strength of the evidence assessed during the meta-analyses according to the GRADE criteria. In each meta-analysis for the head and neck angles, the quality of evidence was very low. No significant inconsistencies were found during the post-hoc subgroup analysis for neck angle, although the very low quality of evidence did not change due to downgrading to very low quality for imprecision, indirectness, and publication bias.

## 4. Discussion

The current systematic review investigated whether the use of a lumbar roll reduced FHP while sitting among individuals with or without musculoskeletal disorders through a meta-analysis of RCTs and cross-sectional studies. The meta-analyses demonstrated a statistically significant reduction in head extension and neck flexion angles, indicating a reduction in FHP. However, the quality of evidence determined using the GRADE system was found to be very low in each meta-analysis. These findings indicate that the conclusions could change with future high-quality studies, and further RCTs are required before a lumbar roll can be recommended for reducing FHP in clinical practice guidelines.

Both healthy individuals and individuals with musculoskeletal disorders were included in this study. However, no participants had spinal symptoms. Thus, whether a reduction in FHP occurs with the use of a lumbar roll among individuals with spinal pain remains unknown. Furthermore, this study synthesized data on neck and head postures immediately after the use of a lumbar roll. Only one study [11] investigated the effect of time on neck and head postures. Furthermore, only one study [11] included a functional task (typing) during measurement. Therefore, possible future research designs could include investigating direct evidence concerning whether the use of a lumbar roll during functional and prolonged tasks while sitting can reduce neck pain, stiff neck, and headaches among symptomatic populations and whether a lumbar roll could be useful in preventing the development of work-related musculoskeletal disorders in the upper body.

The five studies included in the meta-analysis on neck angle had high heterogeneity [9,10,11,12,26]. As such, a subgroup analysis excluding one RCT [26] whose SMD values seemed far larger than those in the other four studies [9,10,11,12] was conducted. However, the results were not different from those of the meta-analysis, with the same quality of evidence. The RCT included individuals with a cranio-vertebral angle <51°, a finding consistent with individuals having FHP and neck pain [26]. Therefore, the magnitude of the reduction in FHP with the use of a lumbar roll might have been greater among symptomatic populations than healthy individuals. A recent systematic review and meta-analysis concluded that the magnitude of FHP was negatively correlated with neck pain intensity and disability [27]. The use of a lumbar roll while sitting during daily activities may be a promising approach for preventing symptom aggravation among patients with neck pain, although clinical assessments for optimal posture should be performed first [28].

The current study has three limitations. The first limitation is the reduction of the quality of evidence level by including RCTs and cross-sectional studies in a meta-analysis. The GRADE level can change from very low to low in the neck flexion angles by limiting the inclusion of the two RCTs [11,26] only. However, such a limited inclusion does not allow meta-analysis on the head angle, and discussions from the findings become difficult. We believe that there is no change in the clinical message from the findings that further RCTs are required before a lumbar roll can be recommended for reducing FHP regardless of the inclusion of cross-sectional studies in the meta-analysis. The second limitation is that we did not impose limitations on included studies based on the type of lumbar roll or specific location over the lumbopelvic region. Accordingly, two studies [9,10] used the Original McKenzie Lumbar Roll (OPTP, Minneapolis, MN, USA), whereas the others [11,12,26] used different lumbar rolls. Three studies applied the lumbar roll over the L3–L4 level [9,10,11], one applied it over L1–L5 [11], and one applied it over L2–L4 [26]. Therefore, the type of lumbar roll or lumbar roll location effective in reducing FHP remains unknown. Future studies from the perspective of ergonomics are required to identify the ideal shape and location of a lumbar roll that will help reduce FHP. The third limitation is that we included the three databases only and we did not include the grey literature. However, we believe that the conclusions in the current study would not change dramatically by adding other databases.

## 5. Conclusions

The current meta-analyses demonstrated that the use of a lumbar roll changed neck and head alignments while sitting, which promoted a reduction in FHP. However, the quality of evidence of the findings was limited to very low. Further studies including symptomatic populations are also warranted to understand whether a lumbar roll could be a useful aid to manage musculoskeletal disorders in the upper body.

## Figures and Tables

**Table 1:** Summary of the five studies included in the meta-analysis.

Study, Corresponding Author Responded or Not or Not Contacted, and the Source of Funding	Design	Participants (*N*, Important Eligibility Criteria, Age, Gender)	Interventions (Lumbar Roll, Backrest Angle)	Comparisons (Measurement Time Points)	Outcomes (Measures, Measurement Tools, Other Outcome Measures Not Included in the Current Review)
Yeoung-Sung [11] Not responded Not described	Randomized controlled design	Total: *N* = 36 Healthy male students Control group (*n* = 12) Thoracic support group (*n* = 12) Lumbar support group (*n* = 12) Age: 26.2 ± 2.7 years Gender: 36 men	Air-mesh and high elastic urethane materials (39 cm wide, 32 cm long, and 8 cm thick) Backrest angles of 90–100°	Immediately after using the lumbar roll while visual display terminal work 20 min after using the lumbar roll while visual display terminal work	Neck: Cranio-vertebral angle ^1^ Measurement tool: Digital image analysis Other objective measures that were not included in this review: Angle between a line from the spinous process of C7 through the tragus of the ear and a line from the tragus of the ear through the eye
Moon [26] Not contacted Not described	Randomized controlled design (cross-over)	Total: *N* = 20 Individuals with the cranio-vertebral angle < 51° Age: 26.6 ± 3.8 years Gender: 10 women and 10 men	Lumbar lordosis assistive support (Chiropractic cushion, Balancecord Inc., Republic of Korea) at L2–4 level Backrest angles of 90°	Immediately after using the lumbar roll while relaxed sitting	Neck: Cranio-vertebral angle ^1^ Measurement tool: Digital image analysis Other objective measures that were not included in this review: Muscle tone, stiffness, and viscoelasticity of the upper trapezius muscle in a sitting position
Horton [10] Not contacted No funding	Before–after design (Quasi-randomized controlled design)	Total: *N* = 30 Healthy males Age: 21.7 ± 3.3 years Gender: 30 men	McKenzie lumbar roll (length (28 cm), diameter (13 cm), and foam density (28 kg/m^3^)) Backrest angles of 90°, 100°, and 110°	Immediately after using the lumbar roll while relaxed sitting	Neck: Cranio-vertebral angle ^1^ Measurement tool: Image analysis Other objective measures that were not included in this review: None
Seung-Hyun [9] Not contacted Not described	Before–after design (Quasi-randomized controlled design)	Total: *N* = 20 Healthy individuals Age: 71 ± 3.6 yearsGender: 1 man, 19 women	McKenzie lumbar roll (length: 28 cm, diameter: 11 cm) Backrest angles of 90°	One minute after using the lumbar roll while watching a TV program on a visual display	Head: The angle between a horizontal line through the tragus of the ear and a line from the tragus of the ear through the eye Neck: Cranio-vertebral angle ^1^ Measurement tool: Image analysis Other objective measures that were not included in this review: None
Majeske [12] Not contacted Not described	Before–after design (Quasi-randomized controlled design)	Total: *N* = 19 Healthy individuals Age: 27.7 ± 5.8 years Gender: 10 women and 9 men	Body Therapeutics at L3 level Backrest angles of 105°	Immediately after using the lumbar roll while relaxed sitting	Head: The angle between a horizontal line through the tragus of the ear and a line from the tragus of the ear through the eye Neck: The angle between a horizontal line through the acromion and a line from the acromion through the tragus of the ear Measurement tool: Analog image analysis Other objective measures that were not included in this review: Angles of trunk, pelvis, upper arm and forearm, and sitting height

^1^ The angle between a horizontal line through the spinous process of C7 and a line from spinous process of C7 through the tragus of the ear.

**Table 2:** Methodological quality of the five studies using the modified McMaster Critical Review Form for Quantitative Studies (≥9/16).

Studies	Criterion No.																Total
	1	2	3	4	5	6	7	8	9	10	11	12	13	14	15	16	
Yeoung-Sung [11]	1	1	1	0	1	0	0	1	1	1	1	1	1	1	1	1	13
Moon [26]	1	1	0	0	1	0	1	1	1	1	1	1	1	1	1	1	13
Horton [10]	1	1	0	0	1	0	0	1	1	1	1	1	1	1	1	1	12
Seung-Hyun [9]	1	1	0	0	1	0	0	1	0	1	1	1	1	1	1	1	11
Majeske [12]	1	1	0	0	1	0	1	1	0	0	1	1	1	1	1	1	11

Criterion 1: Purpose, Criterion 2: Literature review, Criterion 3: Study design, Criterion 4: Blinding, Criterion 5: Sample description, Criterion 6: Sample size, Criterion 7: Ethics and consent, Criterion 8: Validity of outcome, Criterion 9: Reliability of outcome, Criterion 10: Intervention description, Criterion 11: Statistical significance, Criterion 12: Statistical analysis, Criterion 13: Clinical importance, Criterion 14: Conclusions, Criterion 15: Clinical implications, Criterion 16: Study limitations. Score 1: Satisfactory, Score 0: Unsatisfactory. A higher score indicates better methodological quality.

**Table 3:** Quality of the evidence using the Grading of Recommendations Assessment, Development, and Evaluation system.

Quality Assessment						Summary of Findings		
No. of Studies	Risk of Bias	Imprecision	Inconsistency	Indirectness	Publication Bias	No. of Participants (with a Lumbar Roll/without a Lumbar Roll)	Pooled Standardized Mean Difference (95% Confidence Intervals)	Quality of Evidence
Neck angle								
5	No serious limitation due to only studies with acceptable methodological quality, do not downgrade (low quality)	Very serious imprecision due to very limited sample sizes, rate down one level (i.e., from low to very low quality)	Serious inconsistency due to statistically significant evidence of heterogeneity (*p* < 0.001), rate down one level (i.e., from low to very low quality)	Serious indirectness due to inclusion of not completely the same measurements, rate down one level (i.e., from low to very low quality)	Likely due to available evidence from several small studies, rate down one level (i.e., from low to very low quality)	101/101	0.77(0.11 to 1.43)	⊕〇〇〇 Very Low
Head angle								
2	No serious limitation due to only studies with acceptable methodological quality, do not downgrade (low quality)	Very serious imprecision due to very limited sample sizes, rate down two levels (i.e., from low to very low quality)	No serious inconsistency, do not downgrade (low quality)	Serious indirectness due to inclusion of clinically different populations, rate down one level (i.e., from low to very low quality)	Likely due to available evidence from several small studies, rate down one level (i.e., from low to very low quality)	39/39	0.47(0.02 to 0.92)	⊕〇〇〇 Very Low

## Data Availability

Data sharing is not applicable to this article.

## Supplementary Materials

The following are available online at https://www.mdpi.com/article/10.3390/ijerph18105171/s1, Table S1: Search terms for EMBASE, MEDLINE, and the Cochrane Library, Table S2: List of the 31 included studies in the full-text review.
