# Supplementary material for: Lumbar Roll Usage While Sitting Reduces the Forward Head Posture in Healthy Individuals: A Systematic Review with Meta-Analysis
Source: Int J Environ Res Public Health. 2021 May 13;18(10):5171. doi: 10.3390/ijerph18105171 (PMC8152998; doi:10.3390/ijerph18105171)
Supplement: Supplementary file 1 [file ijerph-18-05171-s001.zip › Table S1.pdf]

Table S1. Search terms for EMBASE, MEDLINE, and the Cochrane Library.

#1 back: ta OR back: ab
#2 lumbar: ta OR lumbar: ab
#3 pelvic: ta OR pelvic: ab
#4 sacral: ta OR sacral: ab
#5 #1 OR #2 OR #3 OR #4
#6 pillow: ta OR pillow: ab
#7 support: ta OR support: ab
#8 roll: ta OR roll: ab
#9 #6 OR #7 OR #8
#10 neck: ta OR neck: ab
#11 cervical: ta OR cervical: ab
#12 head: ta OR head: ab
#13 #10 OR #11 OR #12
#14 #5 AND #9 AND #13
