# Supplementary material for: Lumbar Roll Usage While Sitting Reduces the Forward Head Posture in Healthy Individuals: A Systematic Review with Meta-Analysis
Source: Int J Environ Res Public Health. 2021 May 13;18(10):5171. doi: 10.3390/ijerph18105171 (PMC8152998; doi:10.3390/ijerph18105171)
Supplement: Supplementary file 1 [file ijerph-18-05171-s001.zip › Table S2.pdf]

Table S2. List of the 31 included studies in the full-text review.

- Excluded through full-text inspection (N = 26)
  - Due to criterion 1: No assessment of head or neck alignment or no use of a lumbar or pelvic support (n = 20) [1,2,3,4,5,6,7,8,9,10,11,12,13,14,15,16,17,18,19,20]
  - Due to criterion 3: Use of interventions other than a lumbar roll (n = 1) [21]
  - Due to criterion 4: Comparisons two different chairs (n = 5) [22,23,24,25,26]

- Included through full-text inspection (N = 5) [27,28,29,30,31]

1. Swartz, E.E.; Nowak, J.; Shirley, C.; Decoster, L.C. A comparison of head movement during back boarding by motorized spine-board and log-roll techniques. *J Athl Train* **2005,** *40*, 162-168.
2. Rempel, D.; Barr, A. A universal rig for supporting large hammer drills: reduced injury risk and improved productivity. *Saf Sci* **2015**, *78*, 20-24.
3. Menotti, F.; Labanca, L.; Laudani, L.; Giombini, A.; Pigozzi, F, Andrea, M. Activation of neck and low-back muscles is reduced with the use of a neck balance system together with a lumbar support in urban drivers. *PLoS ONE* **2015**, *10*, e0141031-e0141031.
4. Lopez-Valdes, F.J.; Forman, J.L.; Ash, J.H.; Kent, R.; Alba, J.J.; Segui-Gomez, M. Assessment of a head support system to prevent pediatric out-of-position: an observational study. *Ann Adv Automot Med* **2013**, *57*, 297-310.
5. Leinonen, V.; Kankaanpää, M.; Vanharanta, H.; Airaksinen, O.; Hänninen, O. Back and neck extensor loading and back pain provocation in urban bus drivers with and without low back pain. *Pathophysiology* **2005**, *12*, 249-255.
6. Truszczyńska, A.; Lewkowicz, R.; Truszczyński, O.; Rąpała, K.; Wojtkowiak, M. Back pain in Polish military helicopter pilots. *Int J Occup Med Environ Health* **2012**, *25*, 258-264.
7. Alperovitch-Najenson, D.; Katz-Leurer, M.; Santo, Y.; Golman, D.; Kalichman, L. Upper body quadrant pain in bus drivers. *Arch Environ Occup Health* **2010**, *65*, 218-223.
8. Kingma, I.; van Dieën, J.H. Car driving with and without a movable back support: Effect on transmission of vibration through the trunk and on its consequences for muscle activation and spinal shrinkage. *Ergonomics* **2009**, *52*, 830-839.
9. Lange, B.; Nielsen, R.T.; Skejø, P.B.; Toft, P. Centrifuge-induced neck and back pain in F-16 pilots: a report of four cases. *Aviat Space Environ Med* **2013**, *84*, 734-738.
10. Kim, H.J.; Lenke, L.G.; Oshima, Y.; Chuntarapas, T.; Mesfin, A.; Hershman, S.; Fogelson, J.L.; Riew, K.D. Cervical lordosis actually increases with aging and progressive degeneration in spinal deformity patients. *Spine Deform* **2014**, *2*, 410-414.
11. Sovelius, R.; Oksa, J.; Rintala, H.; Siitonen, S. Neck and back muscle loading in pilots flying high G(z) sorties with and without lumbar support. *Aviat Space Environ Med* **2008**, *79*, 616-619.
12. Kuster, R.P.; Bauer, C.M.; Oetiker, S.; Kool, J. Physiological motion axis for the seat of a dynamic office chair. *Hum Factors* **2016**, *58*, 886-898.
13. Kawoosa, A.A.; Wani, I.H.; Basit, S.; Dar, F.A.; Mumtaz, E.U.; Eafiq, M. Reconstruction of unstable hips with Ilizarov technique. Role of pelvic support and distal lengthening realignment osteotomy. *Ortop Traumatol Rehabil* **2015**, *17*, 481-487.
14. Horodyski, M.; Conrad, B.P.; Del Rossi, G.; DiPaola, C.P.; Rechtine, G.R. 2nd. Removing a patient from the spine board: is the lift and slide safer than the log roll? *J Trauma* **2011**, *70*, 1282-1285.
15. Sawada, N.; Hatta, T.; Kishigami, H.; Shimizu, M.; Yoda, T.; Goda, H. The effect of a newly developed wheelchair with thoracic and pelvic support on cervical movement and muscle activity in healthy elderly women. *European Geriatric Medicine* **2015**, *6*, 286-290.
16. Vink, P.; Douwes, M.; van Woensel, W. Evaluation of a sitting aid: the back-up. *Appl Ergon* **1994**, *25*, 170-176.
17. Goda, H. The research of effect in the novel back support set for elderly individuals. Available online: https://upload.umin.ac.jp/cgi-open-bin/ctr_e/ctr_view.cgi?recptno=R000027418 accessed on Aug 28 2016)
18. Kuo, Y.R.; Fang, J.J.; Wu, C.T.; Lin, R.M.; Su, P.F.; Lin, C.L. Analysis of a customized cervical collar to improve neck posture during smartphone usage: a comparative study in healthy subjects. *Euro*

Spine J **2019**, *28*, 1793-1803.
19. Barnes, G.R.; Rance, B.H. Head movement induced by angular oscillation of the body in the pitch and roll axes. *Aviat Space Environ Med* **1975**, *46*, 987-993.
20. Iranzo, S.; Piedrabuena, A.; Iordanov, D.; Martinez-Iranzo, U.; Belda-Lois, J.M. Ergonomics assessment of passive upper-limb exoskeletons in an automotive assembly plant. *Applied Ergonomics* **2020**, *87*, 103120
21. Santamaria, V.; Rachwani, J.; Manselle, W.; Saavedra, S.L.; Woollacott, M. The impact of segmental trunk support on posture and reaching while sitting in healthy adults. *J Mot Behav* **2018**, 50, 51-64.
22. Ukita, A.; Nishimura, S.; Kishigami, H.; Hatta, T. Backrest shape affects head-neck alignment and seated pressure. *J Healthc Eng* **2015**, *6*, 179-192.
23. Ward, J.; Coats, J. Comparison of the BackJoy SitSmart Relief and Spine Buddy LT1 H/C Ergonomic chair supports on short-term neck and back pain. *J Manipulative Physiol Ther* **2017**, *40*, 41-49.
24. Goda, H.; Hatta, T.; Kishigami, H.; Suzuki, A.; Ikeda, T. Does a novel-developed product of wheelchair incorporating pelvic support prevent forward head posture during prolonged sitting? *PLoS ONE* **2015**, *10*, e0142617.
25. Alkhateeb, A.M.; Forrester, B.J.; Daher, N.S.; Martin, B.D.; Alonazi, A.A. Validity and reliability of wheelchair sitting posture measures using Coach's Eye in abled subjects. *Assist Technol* **2017**, *29*, 210-216.
26. Syamala, K.R.; Ailneni, R.C.; Kim, J.H.; Hwang, J. Armrests and back support reduced biomechanical loading in the neck and upper extremities during mobile phone use. *Appl Ergon* **2018**, *73*, 48-54.
27. Moon, J.H.; Jung, J.H.; Hahm, S.C.; Oh, H.K.; Jung, K.S.; Cho, H.Y. Effects of lumbar lordosis assistive support on craniovertebral angle and mechanical properties of the upper trapezius muscle in subjects with forward head posture. *J Phys Ther Sci* **2018**, *30*, 457-460.
28. Seung-Hyun, K.; Yu-Shin, K. Influence of the lumbar spine adjustment using the lumbar roll support on head and neck posture in older adults. *The Journal of the Korea Contents Association* **2011**, *11*, 800-806.
29. Yeoung-Sung, K.; Han-Kyu, P.; Min-Chull, P. Research the effects of thoracic and lumbar support fixtures on forward head posture during visual display terminal work. *J Korean Soc Phys Med* **2016**, *11*, 41-47.
30. Horton, S.J.; Johnson, G.M.; Skinner, M.A. Changes in head and neck posture using an office chair with and without lumbar roll support. *Spine (Phila Pa 1976)* **2010**, *35*, E542-E548.
31. Majeske, C.; Buchanan, C. Quantitative description of two sitting postures. With and without a lumbar support pillow. *Phys Ther* **1984**, *64*, 1531-1535.
